# On a Purely Milk Diet in the Treatment of Diabetes Mellitus, Bright’s Disease, Disease of the Supra-Renal Capsules, Fatty Degeneration, Etc.

**Published:** 1870-11

**Authors:** Arthur Scott Duncan


### On a Purely Milk Diet in the Treatment of Diabetes Mellitus, Bright's Disease, Disease of the Supra-Renal Capsules, Fatty Degeneration, etc.

*By Arthur Scott Duncan, M.D.*

The author continues a dissertation on this extensive subject, through three numbers of the *Lancet*, in which he most strongly advocates the treatment with skim-milk exclusively, of these several troubles, and supports his position with records of cases showing the efficacy of this course.

The milk diet, in the first place, in diabetes, is not objected to by the patient, but is relished, especially at the outset of the treatment when the thirst is intense. In this it is signally unlike, and superior to the exclusively meat diet of Dr. Rollo. The milk diet is as far superior to the meat diet as this was to any other previously used. But it is necessary to be persevered in methodically and exclusively until convalescence is established. If this rule is not followed from the beginning, the result will not be successful.

The use of milk twenty-four hours will produce marked improvement; the quantity and density of the urine fall, thirst and voracious appetite disappear, the skin becomes moist and perspiration is re-established, the troublesome nervous symptoms are abated, and refreshing sleep succeeds to the previous sleepless, restless condition rendered intolerable by the incessant thirst. In the two cases recorded, this rapid improvement was noted. In another case, on the same treatment, at the end of three days and with no other remedy, the urine fell from 23 pints sp. gr. 1038, to 6 pints sp. gr. 1038. In other words, in three days there was a dimunition of 17 pints of urine, and about 24 oz. of sugar. The other prominent symptoms of the disease underwent a like rapid change for the better. The fact that milk exercises a greater influence over diabetes than a purely animal diet, may be explained by the fact that casein, being a primitive albumen, is infinitely superior, as an agent of nutrition, to the albumen of muscle which has been highly organized for an important vital function. Beside the sugar of milk is altogether innocuous in this disease, as has been shown by experiments. It supplies the system with a saccharine, proximate, alimentary principle, equivalent to such as vegetable food affords. The sugar of milk is destined to supply for the nutrition of the young, an equivalent for the amylaceous and saccharine principles of the food of adults. Sugar in moderate quantities is not injurious in diabetes; it cannot be again converted into sugar by any morbid process in the liver or elsewhere, and hence cannot furnish a pabulum for the elaboration of diabetic sugar.

Starch seems to be fuel for the keeping up of morbid action in all save advanced cases, hence its use in any shape should be wholly interdicted, even after recovery and sugar has for a long time been absent from the urine. The necessity of this precaution was shown by the case of one of the patients treated, who before leaving the infirmary, clandestinely partook of large quantities of bread, with the effect of causing the prompt reappearance of sugar in the urine; this, however, subsided in a few days on strictly prohibiting starchy foods.

The success of the milk treatment shows that in this disease it is not necessary to restrict the amount of fluid taken by the patient; the thirst bears a definite relation to the quantity of sugar voided, and subsides as the latter is reduced. All the patients Dr. D. has treated, were kept wholly on the use of skim-milk, until convalescence had been for sometime established, when lean meat and green vegetables were added for dinner.

Two cases are recorded of Bright's disease in which the milk diet was resorted to. In one, the patient had become enormously œdematous and ascitic; the urine was scanty, highly albuminous, and had a specific gravity of 1010. There were found abundant fatty and hyaline tube-casts. The patient was suffering from cephalalgia, nausea, and his countenance was pale, pasty, and puffy.

Five pints of carefully skimmed milk was ordered to be taken in divided doses each day; all other articles of diet were prohibited. A diuretic was likewise ordered, composed of twenty grains acetate of potash and twenty minims tincture of digitalis, to be taken three times a day. This course was pursued for a fortnight, when all traces of albumen in the urine had disappeared, and there was not a trace of dropsy left, neither did any again return; the daily quantity of urine which had been before commencing the treatment five pints, was now reduced to three pints, but its density had increased to 1015.

At this period the diuretic was left off, the milk continued, and a moderate quantity of brown bread allowed at each meal. A tonic of sulphate of quinine and sulphate of iron was ordered. In a month he was dismissed cured. In six months there had been no reappearance of the disease.

In the other case, the patient's condition was very much like the first, only perhaps worse. Exactly the same treatment was prescribed as with the former patient. The improvement was as prompt and positive. But on eating clandestinely starchy foods far beyond the amount allowed, he had a relapse, which brought back his disease with increased severity; his life was almost despaired of, coma began to appear, and death seemed imminent. By the continued use, however, of dractic purgatives, his condition was improved, when the exclusive milk diet was again resorted to, and the patient went on to complete recovery to all appearance.

In about nine months, under bad hygiene at his own home, the disease was gradually reappearing.

A case is recorded of lead-poisoning and Bright's disease, with anasarca and epileptic coma, which had been in progress from, at the beginning, slight pains in the legs and head for six years. The patient had all the symptoms of severe lead-poisoning with albuminous urine. Under an ordinary treatment for the lead-poisoning the case improved in all its nervous manifestations, but the œdema constantly increased, and the deposit from the urine became more abundant. Epileptiform seizures were very frequent and severe. The patient was now placed on an exclusive skim-milk diet, and had twenty grains of acetate of potash three times a day as the only medicine. The case went on to a rapid and complete recovery. These cases were undoubtedly what Virchow calls *parenchymatous nephritis*—the disease being seated chiefly in the glandular epithelium of the uriniferous tubes. The *modus operandi* of the disease seems to be this: The convoluted cortical uriniferous tubules become swollen and distended by their morbid epithelial contents—glued together into solid casts by a fibrinous effusion. The fibrinous capsule of the kidney yielding but slightly to the pressure from within, the secondary capillaries—the efferent vessels of the Malpighian tufts, just external to them, become compressed, and the circulation through them more or less impeded. The blood is consequently dammed up in the primary vessels of the tuft, which are really within the tube and subject to no compression, and there results a constant escape of a large amount of albumen through their walls from the blood. The effect of the drain of albumen from the kidneys, is to impoverish the blood in this direction so much, that from the proportional excess of watery elements the red corpuscles are destroyed, and general dropsy occurs. Now, the first effect of skim-milk, taken to the extent of five or seven pints daily, is to cause a powerful diuretic effect; the effect of the profusion of water is to *flush* the uriniferous tubules, and wash out the material that blocks them up. This relieves the pressure on the secondary capillaries, more and more blood flows through them, and the congestion of the capillaries within the Malpighian tufts is removed, and less and less albumen transudes through their walls. While this beneficial change is going on, healthy epithelium is developed in the tubules which secrete the urinary excrement from the blood.

Milk is superior to other diuretics, because while it removes a large amount of water from the blood, it renews the supply of albumen in this fluid by the amount of this element which is contained in its substance, restoring a healthy condition of the blood, and preventing a *return* of the dropsy.

